# Does male gonopodial morphology affect male-female mating positioning in the livebearing fish *Xenophallus umbratilis*?

**DOI:** 10.1371/journal.pone.0281267

**Published:** 2023-02-02

**Authors:** Mary-Elise Nielsen, Erik S. Johnson, Jerald B. Johnson

**Affiliations:** 1 Department of Biology, Evolutionary Ecology Laboratories, Provo, UT, United States of America; 2 BYU Life Science Museum, Brigham Young University, Provo, UT, United States of America; University of Pretoria, SOUTH AFRICA

## Abstract

*Xenophallus umbratilis* is a freshwater livebearing fish that exhibits unique antisymmetry in the male gonopodium, which terminates in either a dextral or sinistral twist. This asymmetry in the gonopodium suggests that males might exhibit side-biased behavior when interacting with females to mate. We conducted two assays to assess the laterality of male and female mating interactions based on gonopodial morphology. We observed lateralized mating behavior in one test where males with sinistral gonopodial morphology interacted with a single female. However, we did not find lateralized mating behavior in males with dextral gonopodial morphology. We also examined male and female positioning in trials that placed a single female with five males, all with the same morphology. These trials also showed no evidence of lateralized body positioning.

## Introduction

How copulation occurs in sexually reproducing organisms has garnered much attention, and this process is well documented in mammals [1–3], birds [4–6], reptiles [7–9], and some insects [10, 11]. Copulation is not common in fishes, though internal fertilization is known to occur in sharks and rays [12], phallostethids [13], and goodeids [14], but is most often studied in the livebearing poeciliid fishes [15]. Despite a growing body of research in poeciliid fishes, our understanding of the mechanisms and behaviors associated with copulation in these species is incomplete.

Many of the mechanisms and specific details that facilitate sperm transfer and morphological and behavioral traits associated with insemination in poeciliid fishes remain enigmatic [16]. Insemination in poeciliid fishes is facilitated by the male intromittent organ, called a gonopodium, which sometimes features hooks or barbs that may help males anchor to females while swinging the gonopodium into position [16, 17]. Poeciliid males can obtain copulation through courtship displays [18–21] or coercion [22–24]. Hence, how males approach females to mate and how females position themselves relative to males are important components of successful copulation yet are not well understood.

**Fig 1:**
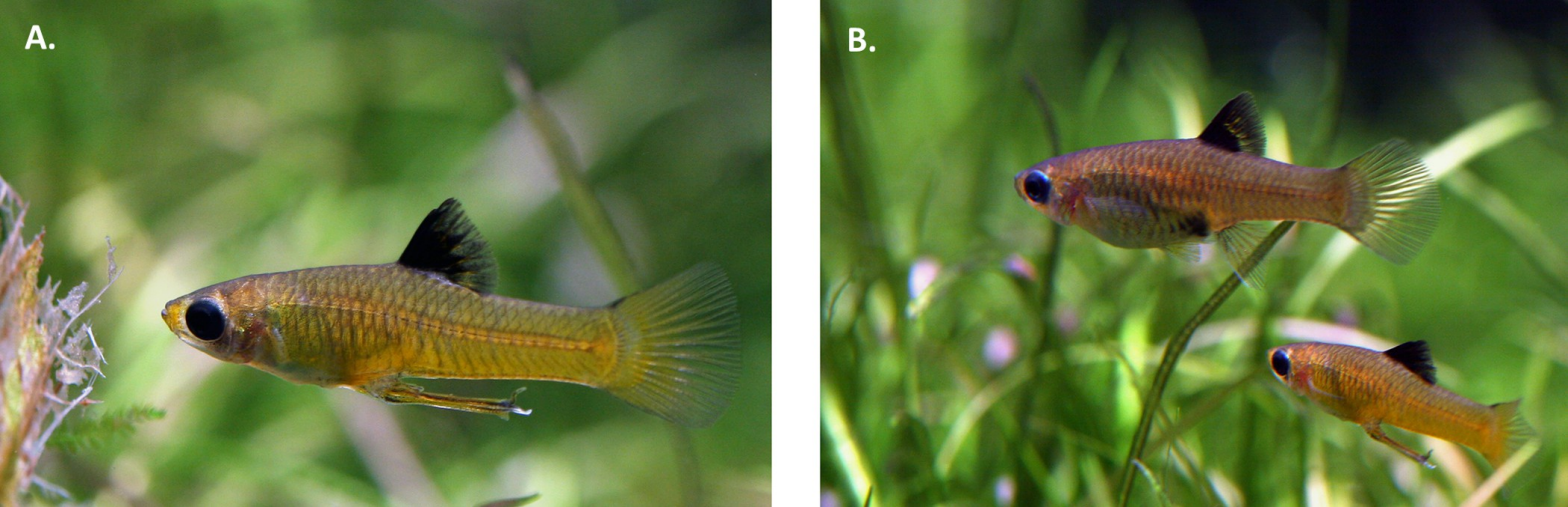
Photographs of male *Xenophallus* and the male gonopodium. (A) Male with a fully developed gonopodium (dextral morph). The hook-like terminus of the gonopodium is visible. (B) Male (bottom) in pursuit of a mature female (top).

Several species of livebearing fishes have males with asymmetric gonopodia, which could create conditions where mating may be side-dependent. Previous work has demonstrated that lateralized gonopodium swinging behavior is correlated with gonopodial morphology in species that exhibit asymmetry or anti-symmetry in the gonopodium [25, 26]. Additionally, in several poeciliid species where the gonopodium is bilaterally symmetrical, no lateral bias in gonopodial swinging behavior was observed [27]. While these studies potentially shed light on male mechanisms in copulation, data that describe how males and females position their bodies relative to each other prior to copulation are essential to understand in livebearing poeciliid fishes. An ideal model to address this would be a species where males have antisymmetrical gonopodia, potentially predisposing them to mating with some directionality. We have found a species of fish that meets this criterion. *Xenophallus umbratilis* (hereafter *Xenophallus*) is a freshwater poeciliid fish native to northern Costa Rica that could be used to understand how males (with asymmetric gonopodia) and females position themselves in a mating context. In this species, the gonopodium is anti-symmetrical: males are either left-handed or right-handed for this trait (Fig 1), with the terminus of the gonopodium having either a sinistral or dextral hook [15]. Moreover, previous work in *Xenophallus* revealed a non-random relationship between lateralized behaviors and gonopodial morphology with mate and predator stimuli [28].

Here, we describe body positioning patterns of male-female mating interactions in *Xenophallus* when females are allowed to interact with either sinistral or dextral morph males. We examine two mating scenarios. In the first scenario, a single male is paired with a single female; in the second scenario, five males of the same morph are paired with a single female. Given previous work showing an association between gonopodial morph and mating directionality in other species [28], it is possible that males preferentially approach one side of a female over the other. Hence, we predict that single males will spend more time on one side of a female than the other side. Further, it is possible that when multiple males are paired with a single female, that males will be more aggressive in their pursuit of the female and she could therefore resist aggressive male copulation events [23, 29]. Hence, we predict that females to mitigate male harassment will position themselves in a way that prevents males from spending as much time on their preferred side.

## Methods

### Study system

**Fig 2:**
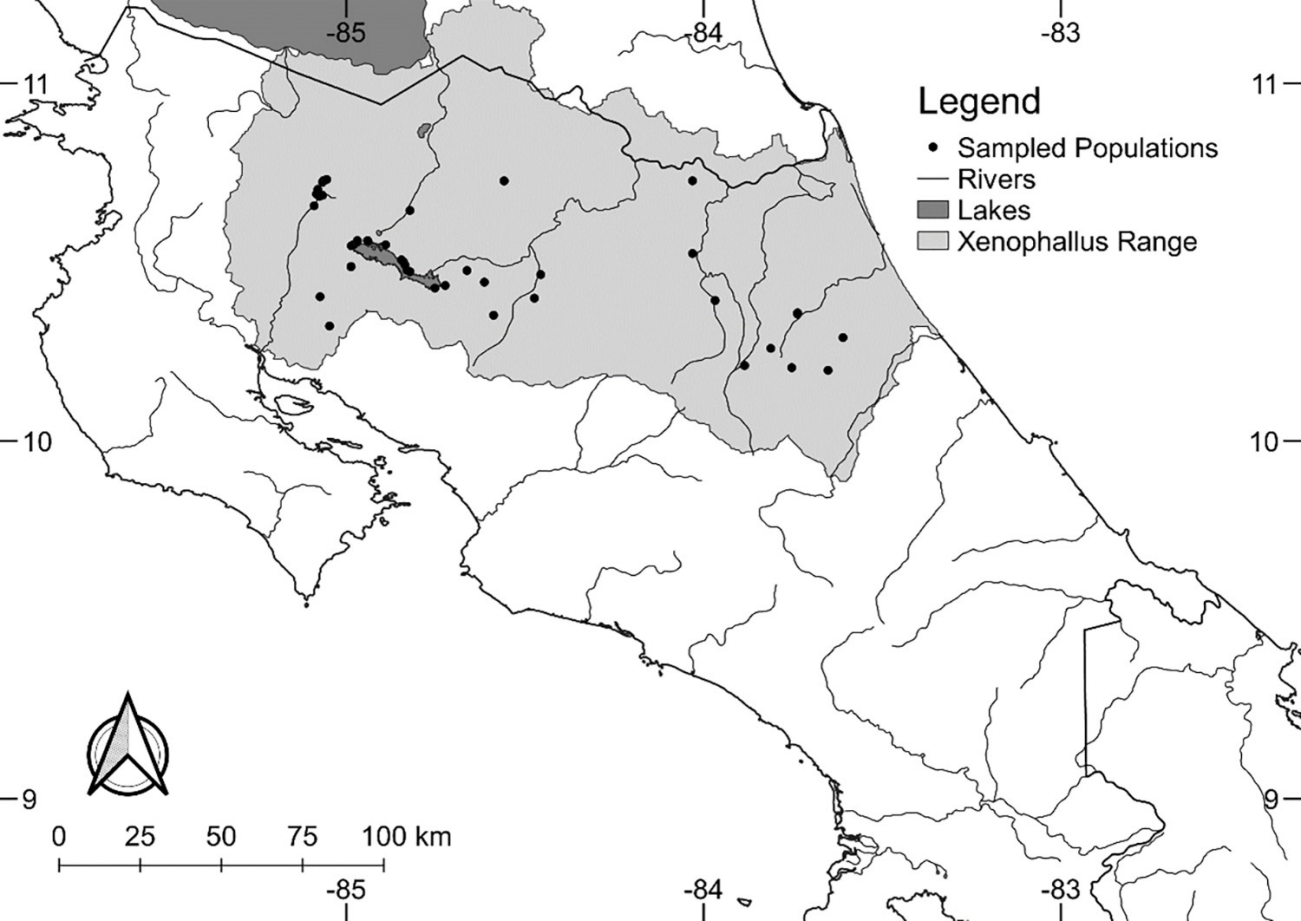
Geographic range of *Xenophallus* in Costa Rica and sampled populations with major river systems. Sampled populations of *Xenophallus* are depicted by black dots, and the species range is shown in gray. Map was produced using program QGIS v3.8.2 [32].

*Xenophallus umbratilis* is a small, tropical fish broadly distributed across northern Costa Rica (Fig 2). This species occurs primarily in small streams and is especially abundant at higher elevations near the headwaters of river drainages [30]. The presence of a gonopodium, a modified anal fin used to inseminate females [16], is a defining characteristic of the sub-family Poeciliinae [17, 31]. *Xenophallus* is unique among Poeciliinae fishes because it exhibits morphological antisymmetry in the male gonopodium.

### Sampling and housing

We collected and transported to BYU 120 live fish (including males and females) from Quebrada La Palma (10.5602333, -84.9407) and 120 more from a second site, Quebrada Chorros (10.476805, -84.6625319), in May of 2019. When we collected in 2019, males from the Quebrada La Palma site were entirely sinistral and males from the Quebrada Chorros site were entirely dextral.

Fish from the La Palma and Chorros populations were housed separately in the lab. Stock tanks were 10-gallons each (42 cm x 27 cm x 22 cm) and held approximately 10–12 fish each, depending on fish size. Within each population, we established several female-only tanks (tanks were the same 10-gallon tanks as the general stock tanks) to house virgin females. Fish were quarantined for one month in the lab, and experiments began two months after the initial quarantine period.

Prior to conducting our behavior assays, we used small tanks to isolate 20 virgin females each from the La Palma (sinistral) and Quebrada Chorros (dextral) populations. Isolated females were of similar size (4.5 ± 0.5 cm in length) and were housed in individual 2-gallon tanks (28 cm x 14 cm x 19 cm), each with a unique identification number (F1-F20), for at least 72 hours prior to testing. We also isolated 15 males each from the Quebrada La Palma and Quebrada Chorros sites. Prior to isolation from the breeding stocks, these males were phenotyped and measured to ensure that all males from Quebrada La Palma were sinistral and all males from Quebrada Chorros were dextral, and that they were of similar sizes (2.75 ± 0.25 cm in length). Within each group of 15 males, individuals were divided into three groups of five males (all same morph) to be used in the Multiple Male assay (see details below). Male groups were rotated out every three trials to avoid male fatigue from prolonged mating pursuit. Following the Multiple Male assay, males were isolated and housed separately before being used in the Single Male assay.

We held all fish under a light regimen of 12-hour light/12-hour dark and fed them twice daily throughout the study. The feeding regimen included fruit flies in the morning followed by TetraMin flakes or crushed krill in the afternoon. For all other variables, we held the fish under common environmental conditions (23–24°C, conditioned water, and gravel substrate and plants in tanks). Following the conclusion of this study, animals were returned to breeding stocks for use in further research.

### Single Male assay

**Fig 3:**
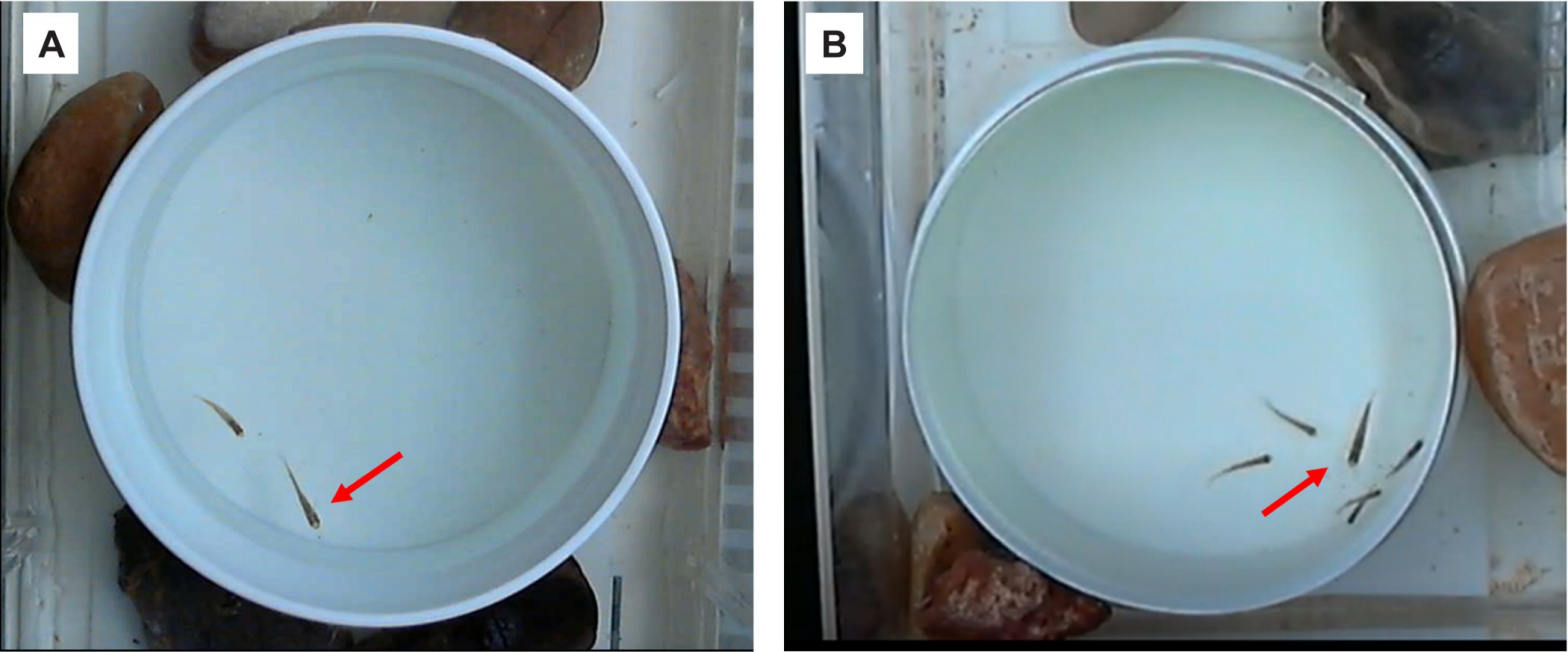
Picture of the experimental tank from the Single Male (A) and Multiple Male (B) assays. Females are indicated by the arrow. All other individuals are males.

In this assay, we paired a single male with a single female. This assay was conducted four months after the Multiple Male assay (see below). We conducted trials in a white, circular tank (Fig 3A) with a camera mounted overhead, in a sound-proof chamber. The circular tank (with no corners to retreat to) eliminated opportunities for females to use the wall to shield against male approaches and the white color made it easier to observe fish as they moved. Two weeks prior to this assay, we separated 15 males each from La Palma and Chorros into individual shoebox tanks with unique identification numbers (M1-M15). Ten females were randomly chosen from breeding stocks and isolated in a 2-gallon tank, with identification numbers F21-F30.

One male (randomly chosen from tanks M1- M15) and one female (from tanks F21-F30) were placed in the test arena (Fig 3A). Fish were acclimated in the tank for 10 minutes and then recorded for 10 minutes. Following recording, we removed the male, and filtered the water through a Tetra Whisper®10i charcoal filter for 10 minutes.

Using the video of each trial, we recorded the average number of times a male was on either side of the female. We scored the position of fish at 30-second intervals. Males and females were distinguished by size. Each video was scored by two people using an established rubric (see details below). In the case of a scoring discrepancy, both scorers reexamined video footage frame-by-frame three seconds before through three seconds after the timestamp of the discrepancy until the final score was agreed upon. Male orientation was recorded as “right” (when a male was on the right side of a female’s body), “left” (when a male was on the left side of a female’s body), or “out” (when males were more than a body’s length from a female or when oriented away from females in a way that did not indicate a mating attempt). This assay was completed first with the sinistral population, followed by the dextral population.

### Multiple Male assay

Female livebearing fishes often resist forced copulation mating attempts from males [29]. Hence, we hypothesized that *Xenophallus* females may avoid forced copulation attempts from males by moving to the side that prevents or disrupts male mating attempts. Without knowing the actual mechanism of sperm transfer in *Xenophallus*, it is not clear if females should move to the left or right of males to avoid insemination. Hence, this assay focused on female-male positioning in a scenario where male coercion behaviors are potentially more aggressive. To test this hypothesis, we examined female behavior in the presence of a group of males, scoring the number of males on either side of a female’s body. We used the same circular, white tank from the first assay, as well as the same camera setup. This assay was conducted four months before the Single Male assay.

We conducted the assay as follows. Fifteen males each were pulled from the La Palma (sinistral) and Chorros (dextral) breeding populations and housed in groups of five. We placed a group of five males with the same gonopodium morphology (sinistral or dextral) in the tank with a single female from the same population (Fig 3B) and allowed all fish to acclimate for 10 minutes. After acclimation, we video recorded interactions between the males and the female for 10 minutes. Following the trial, the female was removed from the tank and returned to her housing tank. We then filtered the water in the testing tank through a Tetra Whisper®10i charcoal filter for 10 minutes. Each group of five males was used in three trials (each time with different females and with a minimum of 10 minutes rest between trials) after which they were replaced by another group of five males. The groups were cycled through in this manner until all females (n = 20) had been tested. Each female was only tested with one group of males.

We scored recordings in a similar manner to that described above in the group assay. Again, the three possible scores were “right” (when the male approached on the female’s right side), “left” (when the male approached on the female’s left side) or “out” (when the male was more than a body’s length from the female or not oriented towards her in a way that indicated a mating attempt). We discarded two trials because we could not readily distinguish the focal female from the males in the recorded video. This assay was completed first with our sinistral population, followed by the dextral population.

### Data analysis

To evaluate whether *Xenophallus* males and females showed a distinct behavioral side bias in mating interactions, we calculated a laterality index (LI), which measured the extent to which an individual demonstrates a side bias to the left or right. We calculated LI using the following equation from Bisazza et al. [33]:

$$LI=\frac{RightPosition-LeftPosition}{RightPosition+LeftPosition}\times 100$$

Positive LI scores indicate a side bias for male positioning on the right of the female. Negative LI scores indicate a side bias for male positions on the left. A LI score of 0 indicates that there is no side bias in positioning behavior. For both assays, we calculated a mean laterality score for the sinistral and dextral test groups and analyzed these results with a two-tailed one-sample t-test to determine if LI scores were significantly different than the null, LI = 0. Statistical analyses were completed in program R [34] and figures were produced using the package ggplot2 [35].

## Results

### Single Male assay

**Fig 4:**
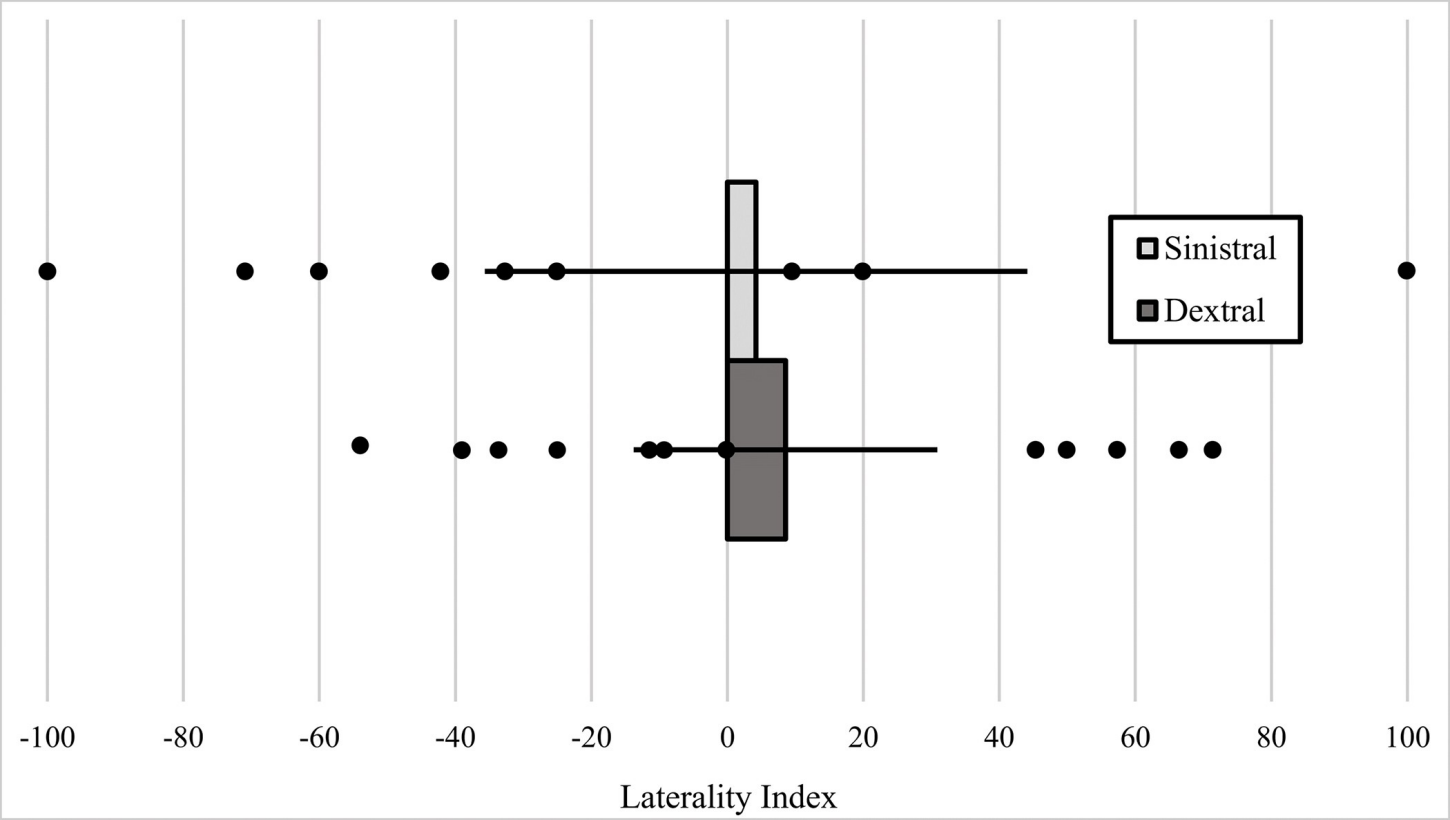
Laterality index scores from our Single Male assay for the sinistral (upper bar) and dextral (lower bar) populations. Each test was comprised of one female and one male, both from the same population. Positive LI scores indicate positioning bias on the right of a female and negative LI scores indicate positioning bias on the left side of a female. A more detailed definition of the LI can be found in the methods. Figure shows a bar chart with error bars depicting ±2 SE. Dots represent LI scores for each individual in this assay.

Fish from our sinistral population did not show significant positioning side bias (LI = 4.209, t = 0.211, df = 14, p = 0.836) (Fig 4). Fish from our dextral population also did not show significant positioning side bias either (LI = 8.561, t = 0.767, df = 13, p = 0.457) (Fig 4).

### Multiple Male assay

**Fig 5:**
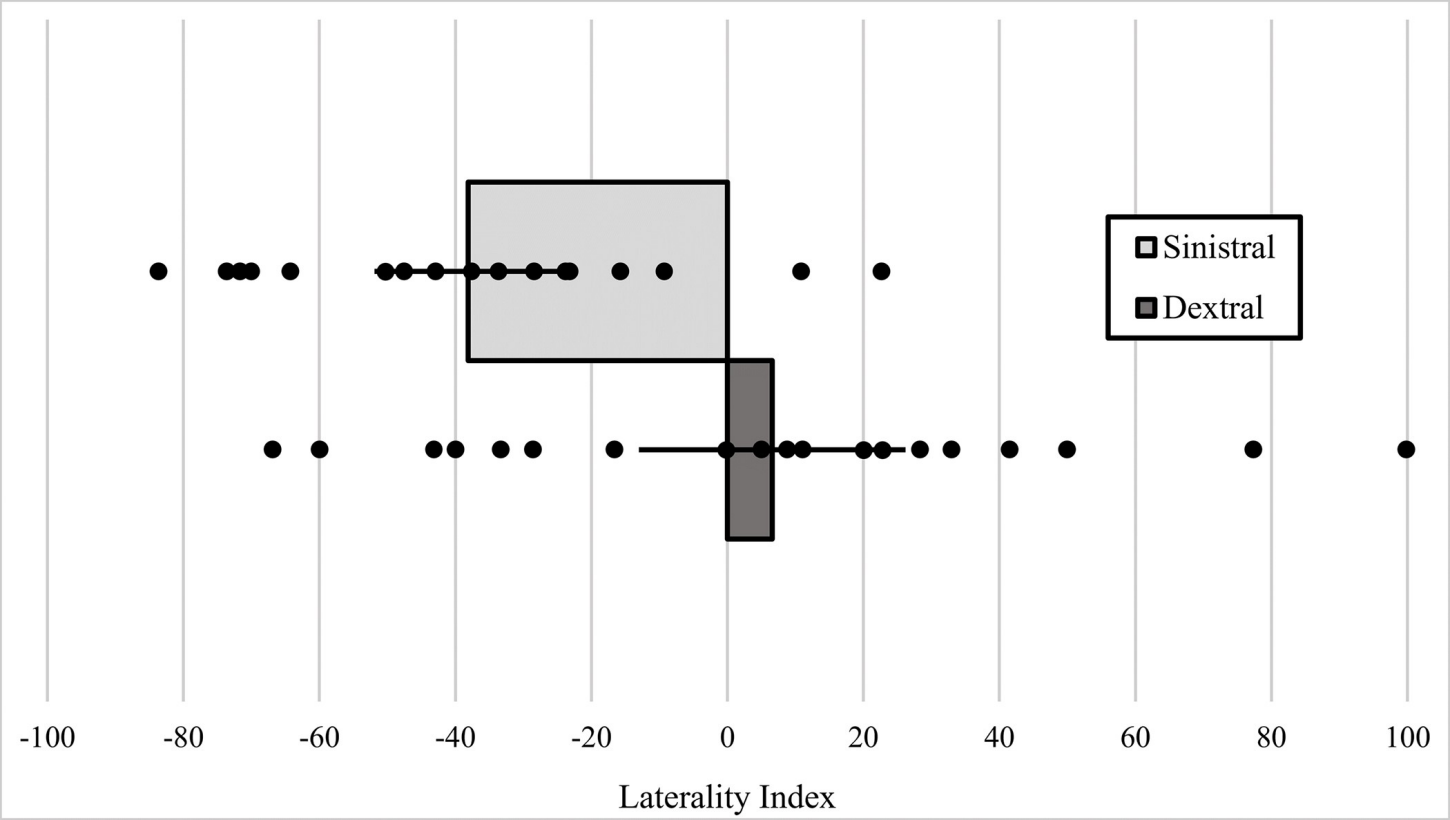
Laterality Index (LI) scores from the Multiple Male assay for the sinistral (upper bar) and dextral (lower bar) treatments. Each test was comprised of one female and five same-morph males, all from the same population. Positive LI scores indicate positioning bias on the right of a female and negative LI scores indicat positioning bias on the left side of a female. A more detailed definition of the LI can be found in the Methods. Figure shows a bar chart with error bars depicting ±2 SE. Dots represent LI scores for each individual in this assay.

Fish from our sinistral population showed a significant left-handed positioning side bias (LI = -38.144, t = -5.543, df = 17, p <0.001), wherein males were most often on the left side of a female (Fig 5). Fish from our dextral population did not demonstrate a statistically significant positioning side bias (LI = 6.590, t = 0.671, df = 19, p = 0.510) (Fig 5).

## Discussion

### Interpreting body positioning

Previous work in other livebearing fishes with asymmetrical gonopodia exhibit side-biased gonopodium swinging [25], which suggests that mating success may be dependent on the side that males are positioned with respect to a female. Hence, we predicted that sinistral and dextral morphed *Xenophallus* males would position themselves differently around females. Puzzlingly, in our study only fish from the La Palma site (composed of sinistral males) demonstrated significantly lateralized positioning behavior, and they only did so in the Multiple Male assay. Males from the Chorros site (composed of dextral males) did not demonstrate lateralized positioning behavior.

It is not clear what could account for the observed discrepancy between dextral and sinistral morph treatments, namely the absence of lateralized behavior in our dextral population. One hypothesis is mutations in asymmetric species may yield antisymmetries if these mutations redirect the genetic “switches” for asymmetrical structures or organs [36]. If this is the case here, it is possible that the dextral morph in *Xenophallus* may be the result of a recent mutation in the species, and that lateralized behavior that corresponds with the dextral morphology has not “caught up” to the more novel phenotype. This lag between morphology and behavior may explain the lack of lateralized positioning in the dextral population. If this is indeed the case, then we would expect body positioning behavior to quickly follow the presence of the dextral gonopodium morph which, according to our work, has not yet occurred. However, in our previous work we found that male gonopodium morphology did accurately predict detour behavior [28] So, in this regard, our results remain somewhat vexing because it appears that males at least approach females with a side-bias as a function of their gonopodial anatomy. An alternative that we have not yet evaluated is that Quebrada Chorros females may in fact be randomly positioning their bodies with respect to males, which would account for our results. Hence, future work that focuses on understanding female positioning behavior while controlling for male morph could help sort out the apparent discrepancy between this study and previous work on detour behavior in this species.

In addition to differences between morphs, we also predicted that side-biases in positioning would vary with the number of males present in an assay. Again, differences in lateralized positioning were only observed in our sinistral population in the Multiple Male assay. We expected to see greater levels of lateralization when more males were present because it could heighten the level of male aggression and in turn cause the female to actively resist aggressive mating attempts [23, 29, 37]. In the Single Male assay, the equal sex ratio may not have provided conditions with enough pressure to generate a strong lateralized positioning behavior in females.

### Potential implications

We made two assumptions in the setup of this study. First, that gonopodium morphology would limit male mating abilities to one side; and second, that females are passive in mating contexts when male harassment is low. Future work should evaluate these assumptions to assess whether male mating success is side-dependent and the extent to which this limits sperm transfer in males. We also recognize that male and female mating interactions are likely not independent of each other in this species, and future work should evaluate the extent to which female behaviors impact male mating success (i.e., what do female avoidance behaviors look like, and under what conditions do females employ these behaviors?).

Additionally, *Xenophallus* may be a valuable model system for understanding how antisymmetrical traits, like the gonopodium, are maintained. This system may be well-suited to investigate negative frequency-dependent selection, one mechanism by which phenotypic variation can be retained through advantages conferred on phenotypically rare individuals [38, 39]. To address negative frequency-dependent selection in this species, future work should evaluate whether gonopodial morphology is heritable and investigate the possibility of rare-male mating advantage in *Xenophallus*.

## Supporting information

S1 Data (XLSX)
